# Effect of processing methods on the chemical composition of *Vitex doniana* leaf and leaf products

**DOI:** 10.1002/fsn3.31

**Published:** 2013-04-02

**Authors:** Frances I. Osum, Thomas M. Okonkwo, Gabriel I. Okafor

**Affiliations:** ^1^ Department of Food Science and Technology Faculty of Agriculture University of Nigeria Nsukka Enugu Nigeria

**Keywords:** Blanching, chemical composition, fermentation, “Uchakoro”, vegetables, *Vitex doniana*

## Abstract

Fresh leaves of *Vitex doniana* were subjected to different food processing methods such as drying, blanching and drying, fermentation of the leaf extract as well as blanching and fermentation of the leaf extract. The proximate composition, mineral and vitamin contents of the fresh and processed leaves were subsequently determined, using standard methods. The results shows that *V. doniana* leaf and products had the following ranges of proximate parameters: 0.07–17.29% protein, 1.85–6.33% fiber, 0.47–6.55% ash, 10.86–95.67% moisture, 0.05–1.29% fat, and 3.61–58.08% carbohydrate. The level of micronutrients detected were generally high and ranged as follows: minerals – Ca (13.38–59.50 mg/100 g), Fe (3.0–18.00 mg/100 g), Na (0.37–1.29 mg/100 g), and vitamins C (1.5–32.98 mg/100 g), A (54.6–3583.26 IU), E (3.11–53.36 mg/100 g), and B_2_ (0.01–9.63 mg/100 g). However, the various processing methods used led to significant (*P* < 0.05) decrease of micronutrients in the extracts, while it caused significant (*P* < 0.05) increase in the protein, fat, and ash content of the oven‐dried sample.

## Practical Applications

This article evaluates the chemical composition (proximate, vitamin and mineral contents) of *Vitex doniana* leaf and leaf products. Results of this study will help to promote utilization and consumption of *V. Doniana*, which is an underutilized plant with great nutritional potentials.

## Introduction

Vegetables, especially leafy vegetables are an important source of vitamins, minerals, fibers, and some essential amino acids (Oyenuga and Fetuga 1975; Grubben 1976; Ihekoronye and Ngoddy 1985). They have high content of water and abundance of cellulose. The cellulose is in a form which although not digested, serves a useful purpose in the intestine as roughages, thus promoting normal elimination of waste products. Vegetables are known to contribute about 90% of vitamins (45% of vitamin A, 27% of vitamin B, 17% of thiamin, and 15% of Niacin), in conjunction with fruits and nuts in the United States diet (Aletor and Adeogun 1995). Vegetables also supply 16% of Mg, 19% of Fe, and 9% of the per capita availability of protein in the United States diet, and their protein are of high quality relative to their content of essential amino acids; other important nutrients supplied by vegetables include folic acid, riboflavin, Zn, Ca, K, and P (Gockowski et al. 2003). In Nigeria, unlike the western world where green leafy vegetables are often consumed in their unprocessed forms, green leafy vegetables are usually subjected to various post harvest treatments, such as drying, blanching, and fermentation (Oboh 2005), in order to improve the taste and modify some of the acids present in the vegetables. The various processing techniques had been reported to alter the nutrients of some commonly consumed plants foods in Nigeria (Oboh and Akindahunsi 2003; Oboh 2005). *V. doniana* (Uchakoro) is a green vegetable which belong to a family called Verbenaceae. *V. doniana* is the most abundant and widespread of the genus occurring in savannah regions. It is a deciduous forest tree of coastal wood land, riverine and lowland forests and deciduous woodland, extending as high as upland grass land. It is readily recognized by its long‐stalked glabrous leaves with the leaflets usually rounded at the apex, though sometimes they may be undented or occasionally have very short triangular tip. The fruits are edible (Keay et al. 1964). As a vegetable**,** the young leaves are collected, boiled overnight, washed in excess water, and then dried. The dried leaves are used to prepare sauces. In folk medicine**,** the leaves are employed in the treatment of stomach pains especially those of a gripping nature and in case of frequent stooling with or without blood in the stool. They are usually chewed, extracted with water or used to prepare yam porridge. It is traditionally known that the efficiency or potency of *V. doniana* leaf extract increases with the intensity of the bitterness, if water extract is of primary interest. In addition, the plant is used in the treatment of mouth ulcers and sores. In this situation, the leaves are washed and chewed after which they are swallowed. The young leaves are used for stock in the Northern part of the country and also as pot herbs (Irvine 1961). In Guinea, a decoration of the pounded roots is used to treat stomach troubles, while in Ivory Coast, the fruits, the stem‐bark, and leaves are used to treat diarrhea and dysentery (Irvine 1961). It has been reported that the exudates of the young leaves are used as a remedy in eye trouble, while the root bark is used to treat rickets and leprosy (Irvine 1961), and infusion of the leaves is used as a relief for cold in Guinea (Dalziel 1955). The objectives of this study were to determine the effect of processing methods on the chemical composition of *V. doniana* leaf and leaf products.

## Materials and Methods

### Materials

Freshly plucked leaves of *V. doniana* were bought from Ogige Market in Nsukka, Enugu State, Nigeria. The leaves were identified to be that of *V. doniana* by a plant Taxonomist in the Department of Botany, University of Nigeria, Nsukka.

### Preparation of *V. doniana* leaf and leaf extract samples

The leaves were destalked, sorted, individually rid of dirt by washing thoroughly under running tap water, drained for 1 h at room temperature (28 ± 2°C), sliced with a sharp knife (2 mm width/strand), divided into portions, and processed to obtain Samples Sample 1, Sample 2, Sample 3, Sample 4, Sample 5 as follows:

#### Sample 1

Steam blanching, oven drying, milling of the dried leaves, and packaging.

Two hundred grams (200 g) of the leaves was steam blanched at 100°C for 8 min. After blanching, the sample was drained, dried at 50°C for 24 h, milled, and packaged in a moisture‐proof nylon bag.

#### Sample 2

Oven drying, milling of the dried leaves, and packaging.

The second portion (200 g) of the leaves was oven dried at 50°C for 24 h, milled to obtain dried leaf powder. (This sample was used as control.)

#### Sample 3

Steam blanching, wet milling, filtration, pasteurization, and packaging.

The third portion weighing 200 g was steam blanched at 100°C for 10 min. After blanching, the sample was wet milled using 250 mL of water (2:2.5) in a Kenwood Blender‐BL300/BL350 and filtered using a sterilized muslin cloth. After filtration, the sample was fermented at room temperature (28 ± 2°C) for a period of 5 days and the extract was pasteurized at 70°C for 30 mins.

#### Sample 4

Fermentation, wet milling, filtration, pasteurization, and packaging.

The fourth portion weighing 250 g was fermented at room temperature (28 ± 2°C) for 5 days. Thereafter, the sample was wet milled using 250 mL of water (2:2.5) in a Kenwood Blender‐BL300/BL350 series and filtered to separate the juice using a sterilized muslin cloth. The extract was bottled and pasteurized at 70°C for 30 min.

#### Sample 5

Raw extract, the raw unfermented extract which served as control, was produced by wet milling the leaves using 250 mL of water (2:2.5) in a Kenwood Blender‐BL300/BL350 series, filtered using a sterilized muslin cloth, bottled, and pasteurized at 70°C for 30 mins.

### Sample analyses

Proximate (protein, crude fibre, ash, moisture, fat, and carbohydrate), vitamins C and E contents were analyzed according to Pearson (1976) methods. Vitamin A content was determined by the method of Jakutowicz et al. (1977), while vitamin B_2_ was determined using Bell's (1994) method. Iron and zinc contents were determined by the method of Vogel (1989), while sodium content was determined by flame photometer method.

### Statistical analysis

Data collected were subjected to one‐way analysis of variance (ANOVA) using SPSS package version 17.0. Means were separated using Duncan's New Multiple Range test (Duncan 1975). A difference was considered to be significant at *P* < 0.05.

## Results and Discussion

Table 1 shows the effect of different processing methods on the proximate composition of *V. doniana* leaf and leaf products. The protein content of the raw leaf (5.82%) was significantly (*P* < 0.05) lower than the protein contents of oven dried and blanched and oven‐dried samples, while extraction and fermentation processes also reduced it significantly (*P* < 0.05). Ihekoronye and Ngoddy (1985) noted that proteins in raw leafy vegetables are usually low, but they have high biological value. The drying process significantly (*P* < 0.05) increased the protein content due to moisture loss, while blanching reduced the protein content probably due to leaching. The drastic decrease in the protein content of the leaf extracts could be due to addition of water during extraction. The ash content of the raw leaf that represents the amount of minerals present in a matter was 3.28%, while that of the extracts were significantly (*P* < 0.05) lower than the powdered samples, due to the dilution effect. There were significant (*P* < 0.05) increase in the moisture content of the extracts 93.78–95.67%, compared with that of the raw leaf. The moisture content of any food is an index of its water activity (Frazier and Westhoff 1978), which is used as a measure of stability and susceptibility to microbial contamination (Scott 1980). Trace amount of crude fiber was detected in the extracts, which could be as a result of dilution effect and filtration process that removed most of the fiber. The powdered samples, however, had the highest fiber contents that ranged from 6.29% to 6.55%. The fiber from vegetables had been reported to serve important function in lowering blood cholesterol concentrations, slowing glucose absorption, weight control, and reducing the risk of colon cancer (Whitney et al. 1996; Garrow and James 1998). The fat contents of these products were quite low, and close to the values (1.2% and 1.8%), reported by Okafor (1995) for dry milled leaves of *Gnetum africanum* and fluted pumpkin, respectively. The fat content of the raw leaf (1.10%) was quite high compared with that of *Solanum lycospersicum* leaf (0.6%) and *Solanum melanaema leaf* (0.4%) (Leung et al. 1968). There were significant (*P* < 0.05) differences in the carbohydrate content of the samples, which were generally low due to added water. The recommended daily intake (RDA) of carbohydrate values for people within 4 years or older, eating about 2000 calories per day is 300 g (Nestle 2000), implying that the carbohydrate values of these products can help to meet the daily requirements if consumed often in reasonable quantity.

**Table 1:** Effect of processing methods on the proximate composition (%) of Uchakoro leaf and its extracts

Treatments	Protein	Fiber	Ash	Moisture	Fat	CHO
Raw leaf	5.85 ± 0.03^c^	1.85 ± 0.01^b^	3.28 ± 0.01^c^	75.00 ± 0.7^c^	1.10 ± 0.01^a^	12.92 ± 0.03^b^
Oven‐dried leaf	17.29 ± 0.01^a^	6.29 ± 0.08^a^	6.42 ± 0.04^b^	11.25 ± 0.54^d^	1.25 ± 0.01^a^	57.50 ± 0.22^a^
Blanched & oven dried	16.89 ± 0.06^b^	6.33 ± 0.04^a^	6.55 ± 0.01^a^	10.86 ± 0.16^e^	1.29 ± 0.04^a^	58.08 ± 0.08^a^
Blanched leaf extract	0.09 ± 0.01^d^	Trace	0.58 ± 0.01^d^	95.67 ± 0.01^a^	0.05 ± 0.01^c^	3.61 ± 0.06^e^
Fermented leaf extract	0.03 ± 0.01^d^	Trace	0.47 ± 0.01^c^	93.78 ± 0.01^b^	0.14 ± 0.00^b^	5.58 ± 0.03^c^
Blanched & fermented leaf extract	0.07 ± 0.01^d^	Trace	0.47 ± 0.01^c^	93.99 ± 0.01^b^	0.15 ± 0.00^b^	5.32 ± 0.06^d^

Values are mean ± SD (*n* = 3). Values within the same column with different superscript are significantly *P* < 0.05 different.

CHO, carbohydrate.

Table 2 shows that drying, blanching, and pasteurization caused significant (*P* < 0.05) reduction in the sodium, calcium, and iron contents of the extracts. Consumption of the extracts would help to meet their recommended daily intakes (RDI) such as 2400 mg for sodium, 1000 mg for calcium, and 18 mg for iron (Nestle 2000). The low level of sodium in all the samples may be supplemented from other sources such as direct addition of salt in diets, which will increase calcium loss in urine if sodium intake is less than 2 g/day. However, this level of sodium may be beneficial for use in sodium restricted diets (0.37–1.29 mg/100 g), as high intakes can contribute to hypertension in some people (Wardlaw and Kessel 2002). The calcium contents of dried leaf powder samples (raw and blanched) that will help to ensure the 20–25% of the daily requirement ranged from 59.50 mg/100 g to 17.29 mg/100 g, while that of pasteurized extracts ranged from 13.38 to 14.33 mg/100 g, respectively. More than 99% of calcium in the body is used as structural components of bones and teeth, which represents about 40% of all the mineral present in the body (Wardlaw and Kessel 2002). The iron content of both leaves compared favorably with the RDI of iron values ranging from 3.00 to 17.29 mg/100 g. This will help to reduce iron deficiency and anemia reported to affect more than 60% of children in Africa (SCN 2010).

**Table 2:** Effect of processing methods on the mineral composition (mg/100 g) of *Vitex doniana* leaf and leaf extract

Treatment	Sodium	Calcium	Iron
Raw leaf	1.29 ± 0.03^a^	51.70 ± 0.03^a^	17.29 ± 0.02^a^
Oven‐dried leaf	1.25 ± 0.01^a^	59.50 ± 0.02^b^	18.00 ± 0.01^a^
Blanched & oven dried	0.94 ± 0.05^b^	17.29 ± 0.02^c^	7.84 ± 0.01^b^
Blanched extract	0.37 ± 0.01^d^	13.38 ± 0.03^d^	3.00 ± 0.17^c^
Fermented leaf extract	0.60 ± 0.01^c^	14.33 ± 0.04^d^	3.50 ± 0.00^c^
Blanched & fermented extract	0.57 ± 0.01^c^	14.30 ± 0.01^d^	3.40 ± 0.04^c^

Values are mean ± SD (*n* = 3). Values within the same column with different superscript are significantly *P* < 0.05 different.

The vitamin C content of the raw and dried leaves in Table 3 ranged from 18.94 to 32.98 mg/100 g, while that of the extracts ranged from 1.56 to 2.16 mg/100 g. The lower level of vitamin C in the oven‐dried samples may be attributed to heat denaturation and oxidation (Oshodi 1992; Yadav and Sehgal 1995). Achinewhu (1983) reported 32–68% loss of vitamin C during handling and processing of some tropical vegetables. Oboh (2005) reported vitamin C losses of 45–82.4% during blanching process. The vitamin B_2_ contents of blanched extracts and blanched/fermented extracts were at trace levels, while the vitamin E values ranged from 3.11 to 53.36 mg/100 g. The significant (*P* < 0.05) decrease between the vitamin E content of the extracts and oven‐dried leaf powder could be as a result of dilution effect. There were significant (*P* < 0.05) differences in the vitamin A content of the samples that ranged from 54.60 to 3583.26 IU. The decrease in the concentration of the vitamin A in oven‐dried leaf powder samples as shown in Table 3 could be due to the activity of lipoxygenase enzymes which degrade carotenoids (Dietz et al. 1988; Booth 1992). Nutrient losses during blanching have been linked to leaching, oxidation of water soluble nutrients, and thermal destruction (Ihekoronye and Ngoddy 1985). Consumption of the leaves of *V. doniana* will help to reduce vitamin A deficiency, which has been estimated to affect about 2.5 million preschool children in Africa, may lead to xerophthalmia, anemia, and weakened host resistance to infection (WHO 2009).

**Table 3:** Effect of processing methods on the vitamin composition of *Vitex doniana* leaf and leaf extract

Treatment	Vitamin A IU	Vitamin B2 (mg/100 g)	Vitamin E (mg/100 g)	Vitamin C (mg/100 g)
Raw leaf	3583.26 ± 4.68^a^	9.13 ± 0.00^a^	53.36 ± 0.03^a^	32.98 ± 0.07^a^
Oven‐dried leaf	3572.30 ± 3.52^b^	9.13 ± 0.00^a^	53.20 ± 0.04^b^	20.83 ± 0.78^b^
Blanched & oven dried	3569.68 ± 2.56^b^	9.06 ± 0.06^a^	53.15 ± 0.04^b^	18.94 ± 0.01^b^
Blanched extract	54.60 ± 0.14^c^	Trace	5.00 ± 0.03^c^	2.16 ± 0.01^c^
Fermented leaf extract	55.54 ± 0.64^c^	0.01 ± 0.00^b^	3.14 ± 0.04^d^	1.56 ± 0.37^c^
Blanched & fermented extract	55.50 ± 0.28^c^	Trace	3.11 ± 0.03^d^	1.52 ± 0.03^c^

Values are mean ± SD (*n* = 3). Values within the same column with different superscript are significantly *P* < 0.05 different.

## Conclusion

The leaf of *V. doniana* has very high nutrient and mineral potentials; however, the processing treatments caused significant (*P* < 0.05) decrease in some of the nutrients.

## Conflict of Interest

None declared.
